# Preclinical ^1^H MRS Study of a Porcine Model Shows Evidence and Mechanisms for Acute Neuronal Injury in Neonatal Cardiopulmonary Bypass (CPB) Surgery

**DOI:** 10.1002/mrm.70364

**Published:** 2026-04-01

**Authors:** Aaron Omon, Meng Gu, Ralph Hurd, Kirk Riemer, Frank Hanley, Daniel Spielman

**Affiliations:** 1 Department of Radiology, Stanford University School of Medicine, Stanford, California, USA; 2 Department of Cardiothoracic Surgery, Stanford University School of Medicine, Stanford, California, USA

**Keywords:** antegrade cerebral perfusion, blood glucose, brain metabolism, cardiopulmonary bypass, circulatory arrest, deep hypothermic circulatory arrest, magnetic resonance spectroscopy, neonatal

## Abstract

**Purpose:**

Congenital heart disease affects 1% of US births, with some infants requiring cardiothoracic surgery under cardiopulmonary bypass (CPB). Optimal surgical parameters to minimize neuronal injury are unknown. We used serial ^1^H MRS in a neonatal CPB porcine model to assess acute neuronal damage and associated injury mechanisms.

**Methods:**

2-week-old piglets (*N* = 26) were placed in a 3T MRI to study brain metabolism during CPB. Dynamic single-voxel ^1^H MRS brain data were acquired while animals underwent CPB via antegrade cerebral perfusion (ACP) or complete circulatory arrest (CA) with findings quantified via ratios to the average total creatine (tCr_ave_). Hypothermic temperatures ranged between 18°C–37°C, and blood glucose (Glc) levels immediately prior to CA ranged from 54 to 700mg/dL. Acute neuronal injury at ~1h post-CA was assessed by decreased N-acetyl aspartate (NAA) and correlated with three potential injury mechanisms: (1) energy failure via pre-CA Glc and phosphocreatine (PCr) loss during CA, (2) reperfusion injury via elevated succinate (Suc) at end of CA, and (3) ammonia toxicity via post-CA glutamine/glutamate (Gln/Glu) levels.

**Results:**

A temperature-dependent NAA/tCr_ave_ decrease was observed for the CA studies, whereas no decreases were seen in the ACP animals. Multiple linear regression analysis revealed that NAA/tCr_ave_ loss was significantly associated with pre-CA brain Glc/tCr (*p* = 0.001) and average PCr/tCr_ave_ levels during CA (*p* = 0.024). NAA/tCr_ave_ losses were not significantly associated with Suc/tCr_ave_ or Gln/Glu changes.

**Conclusions:**

^1^H-MRS in a CPB model identified neuronal injury ~1h post CA, with injury severity correlating with pre-CA Glc/tCr_ave_ levels and loss of PCr/tCr_ave_ during CA.

## 1 | Introduction

Cardiopulmonary bypass (CPB) is necessary for infants with severe congenital heart disease needing major operations on the heart and great vessels, as it maintains oxygen delivery throughout the body during surgery. However, CPB carries a risk of brain injury due to mechanisms such as ischemia, reperfusion injury, hypothermia, inflammation, and hemodilution [1]. Although advances in neonatal cardiac surgery have significantly reduced mortality rates for complex congenital heart diseases (CHD), long-term morbidities, particularly poor neurodevelopmental (NDV) outcomes, remain a concern [2]. In a 2025 study of 2-year neurodevelopmental outcomes in neonates undergoing congenital cardiac surgery with CPB, Mycinski et al. reported non-optimal NDV outcomes in 13.1% of cases [3].

Two CPB methodologies are currently practiced in these circumstances: “Deep Hypothermic Circulatory Arrest (DHCA)” and “Antegrade Cerebral Perfusion (ACP)”. DHCA involves turning the pump off to create a bloodless surgical field, but it also results in no brain perfusion. After the cardiac repair, CPB is restarted, and the patient is then rewarmed and weaned from CPB. Although DHCA is well-studied [4], it has been associated with long-term neurodevelopmental deficits following its use in neonatal cardiac surgery [5–7]. In an ongoing attempt to improve neurocognitive outcomes after open-heart surgery, ACP, wherein the head is continuously perfused with oxygenated blood throughout the entire operation, has evolved as a viable alternative to DHCA. Yet, the neuroprotective advantage of ACP over DHCA remains an ongoing area of investigation [8, 9]. Hence, understanding the specific biochemical mechanisms underlying CPB-induced neuronal injury is crucial for improving patient outcomes.

A piglet model was chosen for this study, as swine are precocial animals with gyrencephalic brains that closely resemble those of humans [10], making them a valuable model system for studying neurodevelopmental outcomes in pediatric patients with congenital heart disease. The translational relevance of piglets is also supported by resting-state brain networks homologous to those observed in humans, as demonstrated by fMRI studies [11]. Furthermore, they have been extensively used to model hypoxic–ischemic injury in pre-, peri-, and post-natal development [12, 13]. For example, Kurth, et al. found regional patterns of neuronal injury after DHCA in a piglet model, suggesting neocortical and hippocampal neurons are selectively vulnerable to death after DHCA [14].

Current CPB brain monitoring tools include electroencephalography (EEG), near-infrared spectroscopy (NIRS), visible light spectroscopy (VLS) [15], and emboli detection and classification (EDAC) [1]. However, the most direct metabolic insights come from preclinical studies using brain microdialysis measurements. For example, using a piglet model, Mavroudis et al. reported cerebral mitochondrial dysfunction and increased lactate and lactate/pyruvate ratios with DHCA based on combined measurements from cerebral microdialysis, blood chemistry, and in vitro assessments of mitochondrial respiration and reactive oxygen species generation [16]. Increased lactate/pyruvate ratios in DHCA-treated swine have also been observed in other studies [17, 18]. Furthermore, microdialysis measurements have shown increased extracellular brain glutamate and glycerol levels associated with ischemic injury [19]. However, more extensive in vivo measurements of brain metabolism during CPB remain challenging as hypothermia affects apoptosis and mitochondrial dysfunction, inflammation, the blood–brain barrier, and free-radical production [20], and reduced or halted cerebral blood flow initiates anaerobic glycolysis, stimulation of inflammatory processes, and other secondary brain insult mechanisms [21].

^1^H-MRS provides unrivaled opportunities to detect subtle CPB-induced brain metabolic changes in vivo, allowing for the quantification of 17–20 metabolites including N-acetyl aspartate (NAA, a neuronal marker), phosphocreatine (PCr, an energy substrate), succinate (Suc, a TCA intermediate), glutamate and glutamine (Glu and Gln, involved in neuronal function and ammonia detoxification), lactate (Lac, a measure of anaerobic metabolism), and glucose (Glc, the brain’s primary energy substrate) [22]. Brain temperature can also be monitored via the chemical shift difference between water and NAA [23]. Although post-operative in vivo ^1^H MRS has been used to evaluate CPB patients [24], the use of dynamic MRS to study brain metabolic changes during CPB surgery in preclinical models has been limited [19, 25]. In contrast to prior imaging studies using methods such as DWI [24, 26–28], which found limited correlations between MRI-detected brain injury from DHCA and long-term outcomes, this study builds upon our single-voxel ^1^H MRS findings to provide a more complete picture of brain cellular health and recovery.

NAA is found almost exclusively within neurons [29], and non-invasive measurement by ^1^H-MRS makes NAA a promising indicator for assessing neuronal viability following multiple brain injuries or insults [30, 31], with decreases in brain NAA concentration being an index for acute neuronal damage [32–34]. According to a review paper by Demougeot et al., reductions in NAA following acute stroke can be considered as a marker of neuronal dysfunction, injury, or loss [30], and a decline in brain NAA has been observed in new-born piglets subjected to hypoxia-ischemia [35, 36]. This early loss of NAA likely reflects the presence of both non-functional and dysfunctional neurons [30]. However, the usefulness of NAA as a quantitative neuronal marker is more limited for chronic injuries due to its redistribution by glial cells and trapping in cellular debris [30].

Glc is the brain’s dominant energy source. Under aerobic conditions, each Glc molecule can be fully catabolized to generate ~32 ATPs, and ATP depletion has been shown to be a driving factor in tissue injury following stroke [37, 38]. Hence, ensuring Glc availability for ATP production during CA is critical for maintenance of cellular function and prevention of cellular injury.

Under normal conditions, creatine (Cre) is synthesized in the brain and is used to form PCr [39]. During ATP depletion, PCr allows ATP synthesis to occur even in the absence of oxygen and glucose [39]. Hence, as O_2_ availability decreases, such as during ischemia, ATP production is driven by PCr stores [39], and fully depleted PCr stores can result in critical energy failure and cellular death.

Elevated Suc during ischemia has been identified as the primary source of ischemic-reperfusion (IR) injury [40–46], which occurs when the tissue blood supply is disrupted and then restored. While reperfusion is essential for survival, it also initiates oxidative damage, cell death, and aberrant immune responses through the generation of mitochondrial reactive oxygen species [47]. We have recently reported that brain Suc is detectable in CPB porcine models, with baseline levels rising during CA and then normalizing upon reperfusion [48].

In addition, we have recently reported on the detection of elevated blood ammonia levels in these models, which are associated with a corresponding increase in brain Gln/Glu ratios [49]. Given the known toxicity of ammonia to the developing brain [50], elevated ^1^H-MRS-detected Gln/Glu ratios may also serve as a potential marker for brain injury.

The overall goal of this study was to use a porcine model of neonatal CPB surgery in combination with dynamic ^1^H brain MRS to assess if acute neuronal damage is detectable during these procedures and, if present, identify the most important injury mechanisms.

## 2 | Methods

### 2.1 | Animal Model and Surgical Protocol

For this study, we targeted a 60min circulatory arrest time with elevated hypothermia temperatures ranging from 18°C to 37°C. Specifically, we conducted serial ^1^H-MRS data from neonatal porcine CPB surgeries [25, 51] to identify metabolic markers of potential brain injury from three independent mechanisms: energy failure, reperfusion injury, and ammonia toxicity. All CPB studies of weaned ~14–21-day-old domestic piglets were conducted under an approved Stanford University Institutional Animal Care and Use Committee protocol. As listed in Table 1, a total of 26 animals were studied in a 3T MRI clinical scanner (MR750, GE Healthcare, Waukesha, WI. USA) using two surgical approaches: complete CA (*N* = 21) and ACP (*N* = 5), both performed with simultaneous ^1^H-MRS and at temperatures ranging from 18°C to 37°C (see Table 1 for complete breakdown of surgery types, associated hypothermic temperatures). Multiple spectra were then acquired from each animal at baseline, during CA, and during recovery; each animal served as its own control. The inclusion of the ACP group was added as an additional control. Specifically, during ACP, the brain is continuously perfused with oxygenated blood; hence, less brain injury was expected. Also listed in Table 1 are the corresponding blood glucose levels, which range from 54 to 700mg/dL (as measured immediately prior to antegrade perfusion in the case of ACP or full circulatory arrest in the case of CA).

A more detailed description of the surgical protocols can be found in [25, 49]. Briefly, after induction of anesthesia, each animal was intubated, placed on a ventilator, and maintained by 1%–2% isoflurane. Cannulas were inserted in both the femoral artery and vein for arterial and venous blood sampling. A median sternotomy was performed, and the major vessels were dissected and mobilized for cannulation and isolation of perfusion to the head. Heparin (300IU/kg) was administered, and the piglets were then transported to the imaging suite and placed in the MRI scanner.

**FIGURE 1 |:**
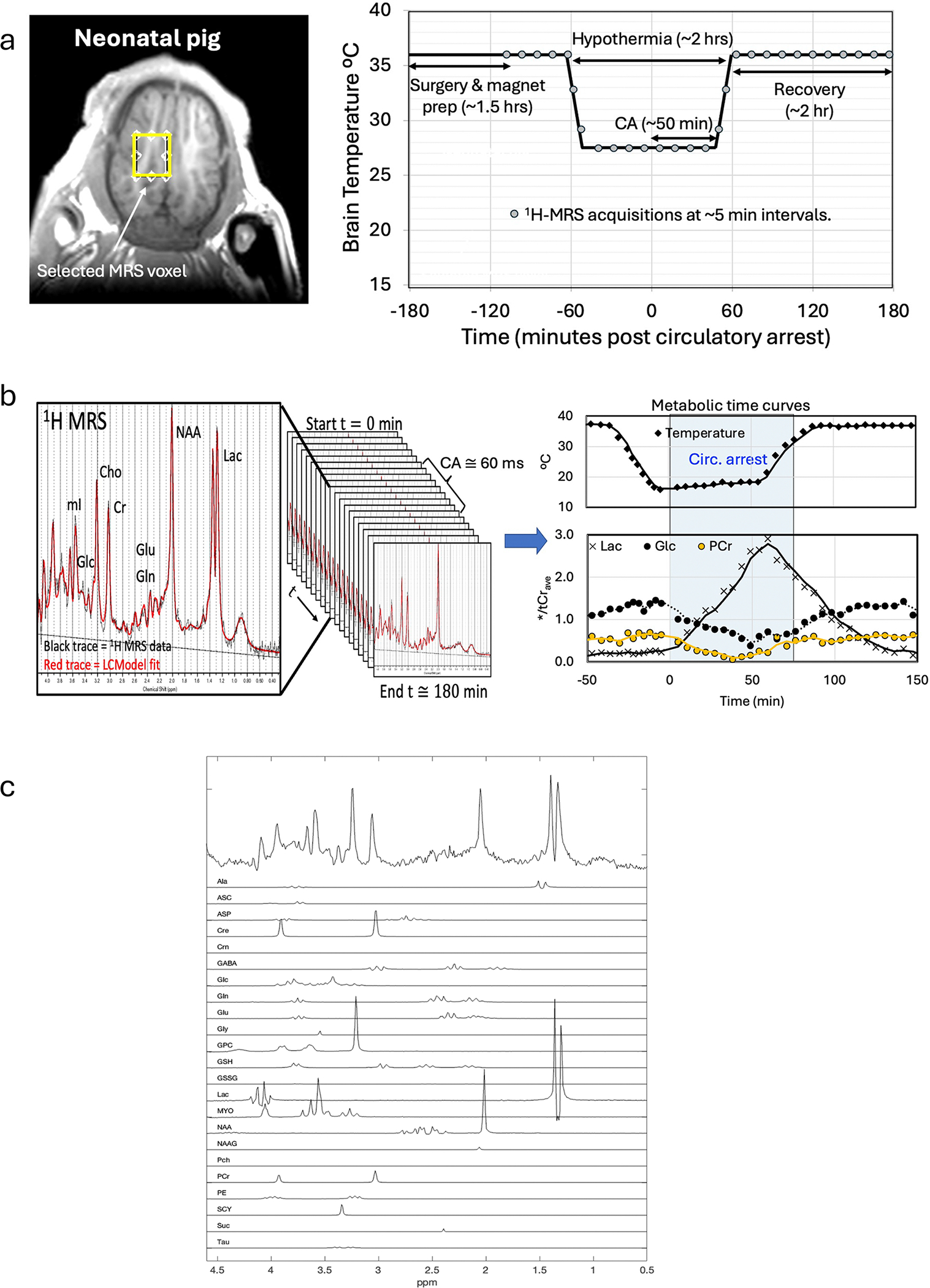
In-magnet CPB porcine study. (a) experimental setup and (b) representative serial ^1^H MRS data from a CA-28°C study, and (c) corresponding metabolic time curves for brain temperature (as measured by the water-to-NAA chemical shift), lactate (Lac/tCr_ave_), glucose (Glc/tCr), and phosphocreatine (PCr/tCr_ave_). ^1^H MRS acquisition parameters: 3T GE MR750 scanner, eight-channel ^1^H RF knee coil, 12 × 12 × 15mm^3^ right midbrain voxel, TR/TE 2000/30s sLASER, 5000Hz spectral bandwidth, 4096 datapoints, 128 averages. Spectral fitting performed using LCModel software [52]. (c) Representative spectrum acquired during circulatory arrest along with the full set of LCModel metabolic basis functions (see [25]). The following metabolites were included in the basis set: Alanine (Ala), ascorbate (ASC), aspartate (ASP), creatine (Cre), creatinine (Crn), GABA, glucose (Glc), glutamine (Gln), glutamate (Glu), glycine (Gly), **g**lycerophosphocholine (GPC), glutathione (GSH), oxidized glutathione (GSSG), lactate (Lac), myo-inositol (MYO), N-acetyl aspartate (NAA), NAAG, phosphocholine (PCh), phosphoethanolamine (PE), scyllo-inositol (SCY), succinate (Suc), and taurine (Tau).

**FIGURE 2 |:**
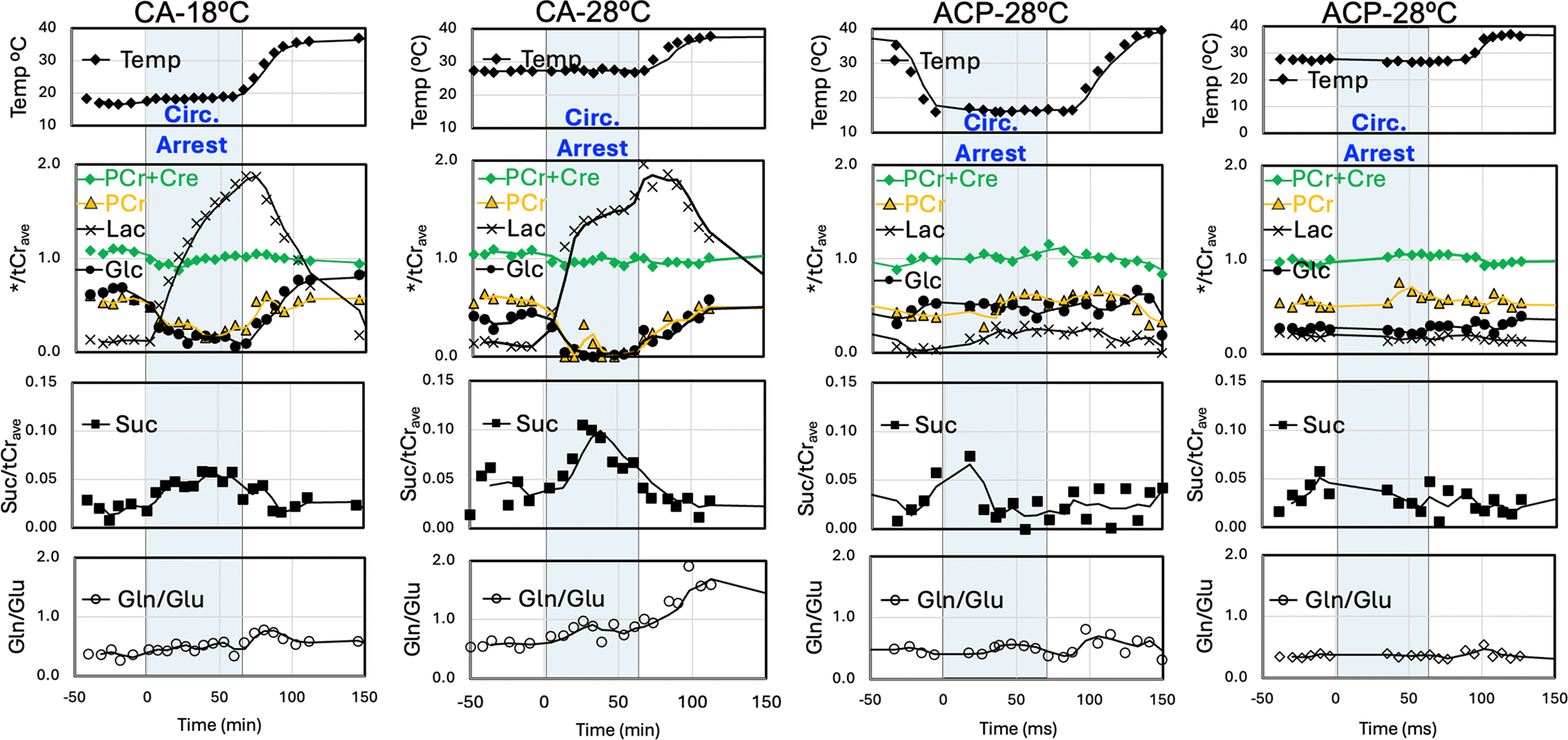
Representative metabolic time courses for porcine CPB studies conducted under four surgical conditions, showing CA-protocol temperature-dependent increased Lac/tCr_ave_, Suc/tCr_ave_, and Gln/Glu along with decreased PCr/tCr_ave_. Minimal changes were noted for ACP. ^1^H MRS acquisition parameters: 3T GE MR750 scanner, eight-channel ^1^H RF knee coil, 12 × 12 × 15mm^3^ right midbrain voxel, TR/TE 2000/30s sLASER, 5000Hz spectral bandwidth, 4096 datapoints, 128 averages.

Within the scanner, the CPB circuit consisted of a roller pump, a membrane oxygenator, ¼” ID tubing from the pump (located in the MR control room), and 3/16” ID tubing from the piglet to ensure unrestricted flow for the extended tubing loop. The circuit was primed with donor pig blood mixed with crystalloid prime solution and filtration concentrated as needed to maintain hematocrit no lower than 30%. CPB was initiated with the aid of vacuum-assisted venous drainage, and 1%–2% Isoflurane was continued on pump. Cooling was initiated at a pump flow of 200mL/kg/min, and hematocrit was maintained at around 30%, with phentolamine administered during the cooling process. Once the nasopharyngeal temperature reached the targeted value, total body perfusion was reduced to 100mL/kg/min, the ascending aorta was then clamped, and cold cardioplegia was administered via the aortic root. For CA protocols, pump flow was stopped for ~60min. For ACP, the head vessels were isolated by cross-clamping the distal arch, left subclavian artery, and left carotid artery, directing flow to the right carotid artery at 40mL/kg/min. At the end of ACP or CA, all clamps were removed, phentolamine was administered, and rewarming was commenced under a temperature gradient of less than 8°C between the arterial blood temperature going to the piglet and the piglet’s core temperature, using a total body flow rate of 200mL/kg/min under alpha-stat blood gas management. Once a temperature of 37°C was achieved, CPB was discontinued. Figure 1 shows the overall experimental setup for a representative CA study, and Figure 2 shows representative metabolic time curves as measured by ^1^H-MRS, with spectra acquired at approximately 5min intervals before, during, and after surgery.

### 2.2 | MRI/MRS Acquisition and Data

#### Processing Protocol

Single-voxel ^1^H MRS was used to target a single 12×12×15mm^3^ voxel encompassing primarily the right cortex but also containing parts of the right hippocampus and internal capsule [53] (see Figure 1a). As previously described in Spielman et al., *NMR Biomed* 2022 [25], serial spectra were initially acquired approximately every 5min using sLASER [54, 55], with TR 2000ms, TE 30ms, 4096 datapoints and 5000Hz spectral bandwidth. For the first 10 animals, each time point consisted of 128 water-suppressed transients along with 8 water-unsuppressed acquisitions plus time to reach a steady state (4:40min:sec total acquisition time). For the subsequent 16 animals, based on an SNR analysis of the prior data, we determined that we could reasonably decrease the number of transients from 128 to 64 for each time point, thereby increasing the overall temporal resolution used to better track metabolic changes throughout the surgery.

Water suppression was achieved using VAPOR, with 5% residual water retained in the water suppressed data by design (used to correct phase and frequency errors in individual acquisitions) and subsequently removed by pure water subtraction [49].

LCModel (version 6.3–1J) software [52] using temperature-dependent basis sets [25] was used to fit each individual spectrum and corrected for polarization changes at different temperatures. Specifically, basis functions were acquired using two sets of sLASER measurements at 37°C and 20°C, which were acquired from each of 23 metabolites by having the chemical phantoms in a temperature-controlled incubator overnight.

Brain temperature at each time point was estimated from the chemical shift of water relative to NAA [23]. Metabolite levels at each time point were computed using the LCModel output values quantified using temperature-dependent basis sets [25] relative to the total tCr = PCr + Cre signal as averaged over each experimental run (tCr_ave_). Further details on separating Cre from PCr signals using ^1^H-MRS at 3T can be found in Hurd et al. [56].

For each pig, metabolite concentration versus time curves were thencomputedasfollows.Serial^1^H-MRSspectrawerefirstgenerated from the LCModel fits starting with initial metabolite values after the animal was stabilized in MRI scanner and extending to 1–1.5h post end-of circulatory arrest and rewarming to standard body temperature (see Figure 2).

Among the 17–20 metabolites detected in these ^1^H-MRS studies [25], we determined that NAA (a marker of acute neuronal injury [30]), Glc, and PCr (the brain’s primary sources of energy via ATP production[37–39]), Suc (elevated Suc being identified as the primary source of ischemic-reperfusion injury [40, 43, 57]), and Gln, Glu, and Lac (identified as important metabolites in potential ammonia toxicity occurring during neonatal CPB surgery [49]) were the most relevant metabolites for assessing neuronal damage from ischemia and associated injury mechanisms.

For all metabolites, temporal curves were computed via a two-point running average of corresponding LCModel spectral peak estimates. Accordingly, we selected the following metrics for assessing changes in NAA/tCr_ave_, PCr/tCr_ave_, Suc/tCr_ave_, and Gln/Glu. Note, the choice of Gln/Glu as a metric, rather than the more common Glx = Glu + Gln, was chosen in order to capture both the increase in Gln accompanied by the decrease in Glu associated with brain ammonia toxicity [49]. The change in NAA/tCr_ave_ was measured by the difference between the NAA/tCr_ave_ value measured at baseline and at the end of each experiment (ΔNAA/tCr_ave_), as computed from an average of last 3 time-points after animal was stabilized in the magnet, but prior (to initiation of cooling) and post-surgery NAA/tCr_ave_ levels (as computed from an average of last 3 time-points acquired 1–1.5h after restoration of blood flow and rewarming of animal to standard body temperature). Brain Glc/tCr_ave_ levels were recorded immediately prior to circulatory arrest. Because the PCr/tCr_ave_ levels during CA for multiple animals fell below robust detection limits we instead choose to use the average value of PCr/tCr_ave_ as measured during CA as a metric. The Suc/tCr_ave_ metric was chosen as the maximum value (which occurred at the end of circulatory arrest for all cases), and the Gln/Glu metric was the maximum Gln/Glu ratio occurring during the post-CA recovery period.

We then performed the following statistical analyses. All ACP studies showed no significant changes in metabolite ratios during the exam, independent of hypothermic temperature. Accordingly, we combined all ACP animals into a single study group. This resulted in the following four animal groups: ACP (*N* = 5 animals), CA-18°C (*N* = 10), CA-28°C (*N* = 9), and CA-37°C (*N* = 2) (see Table 1). Two-sided *t*-tests were performed to assess statistical differences between these groups for ΔNAA/tCr_ave_, average PCr/tCr_ave_, Suc/tCr_ave_ at the end of CA, and maximum Gln/Glu. Linear regressions were then calculated for ΔNAA/tCr_ave_ versus brain Glc/tCr_ave_, blood Glc, PCr,/tCr_ave_ Suc/tCr_ave_, and Gln/Glu.

Finally, to assess which of the metabolic metrics were most strongly correlated with loss of NAA, we performed a multivariate linear regression with output ΔNAA/tCr_ave_ and inputs brain Glc/tCr prior to CA, average PCr/tCr_ave_ during CA, Suc/tCr_ave_ at the end of CA, and maximum Gln/Glu post-recovery.

## 3 | Results

To assess the interpretability of reporting metabolite values relative to tCr_ave_, we first measured temporal changes in tCr values. For all animals, tCr values varied on a point-by-point basis by ±5%, with no statistically significant differences found between tCr values during baseline, circulatory arrest, and recovery periods, independent of hypothermic temperature.

**FIGURE 3 |:**
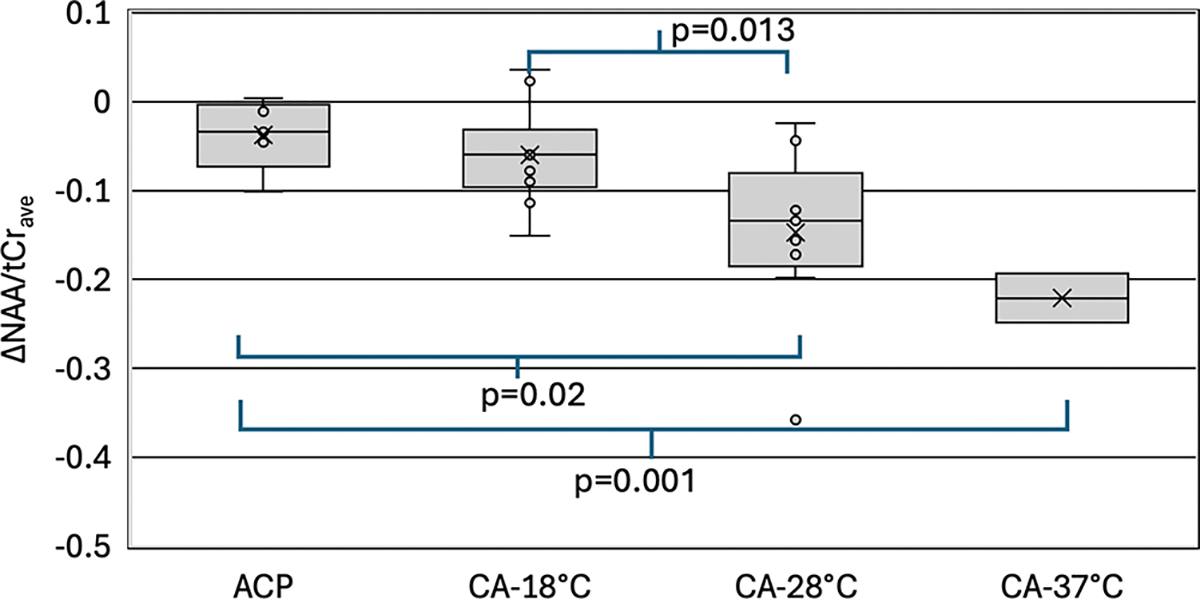
Loss of NAA/tCr_ave_ from baseline to end of in-magnet study (~1h post CA) as a function of surgery parameters. Statistical differences were computed using two-sided paired *t*-tests. In contrast to the CA studies, the ACP studies showed no statistical NAA/tCr_ave_ differences with temperature; hence all ACP data was combined and plotted together.

The average SNR for a 64-transient ^1^H-MRS acquisition was ~25, as reported by LCModel. As shown in Figure 3, a decrease in NAA/tCr_ave_ between baseline (pre-CA) and ~1h post reperfusion was found to be highly correlated with surgical type and temperature (*p*=0.0003). The greatest NAA/tCr_ave_ loss, and thus presumed acute neuronal damage, was seen in the piglets undergoing complete CA at elevated temperatures. In contrast, NAA/tCr_ave_ loss was minimal during the ACP surgeries, with no significant differences between temperature conditions (hence, they are plotted together in Figure 3).

**FIGURE 4 |:**
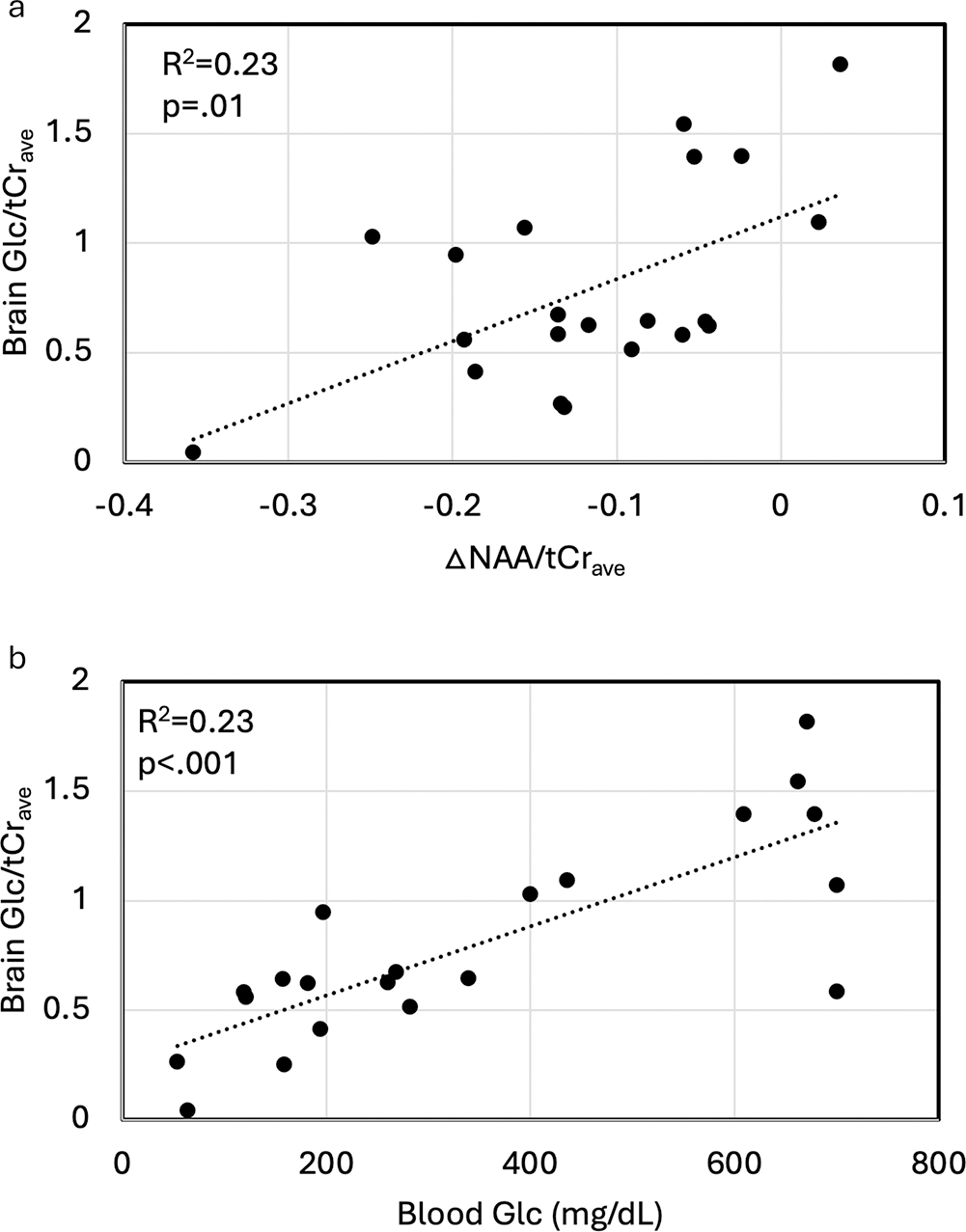
NAA/tCr_ave_ findings. (a) Linear regression of loss of NAA/tCr_ave_ (as measured by difference between brain NAA at start of CA and ~1–1.5h post reperfusion) versus brain Glc/tCr_ave_ at start of circulatory arrest and (b) blood glucose versus brain Glc/tCr_ave_ levels at start of CA.

**FIGURE 5 |:**
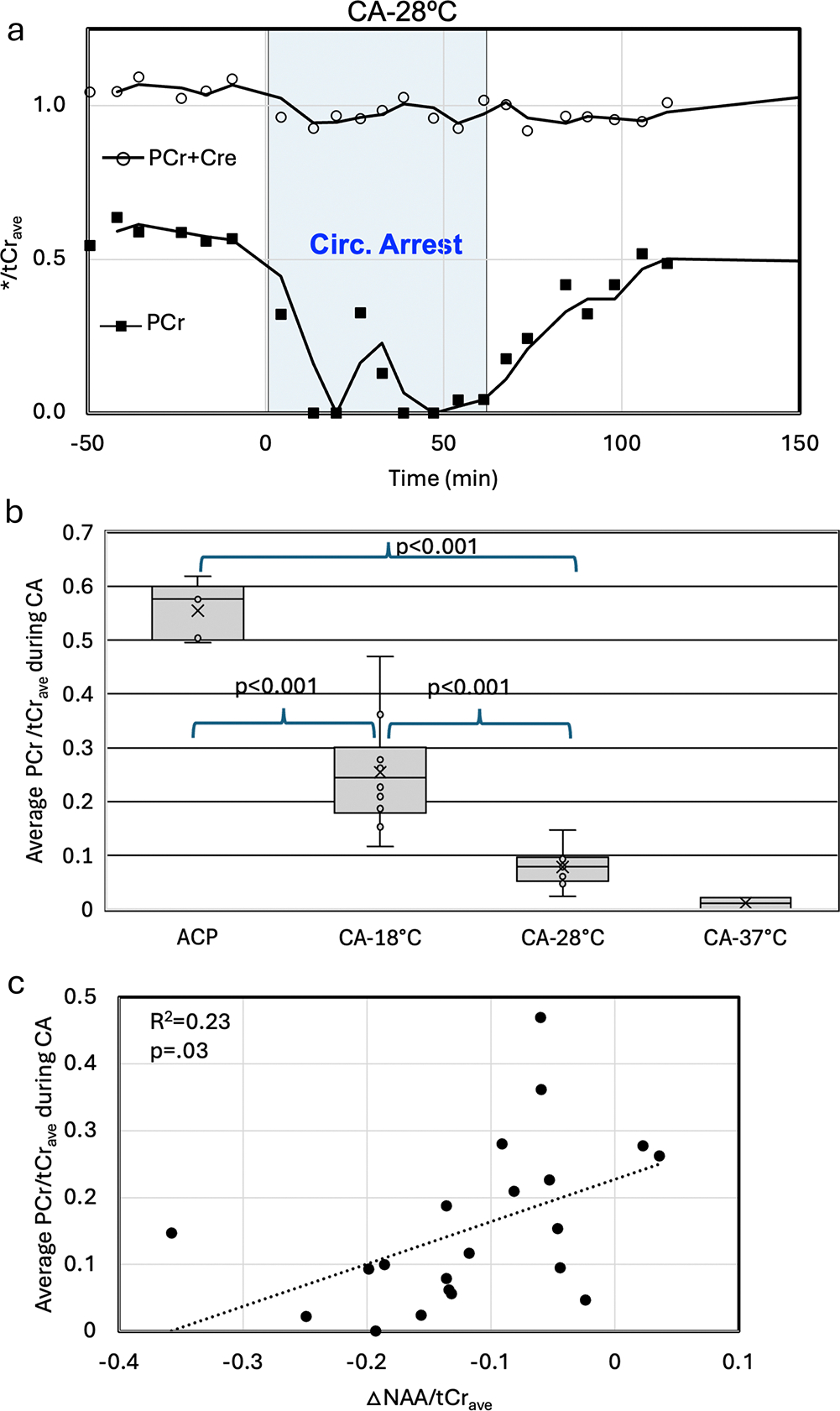
PCr/tCr_ave_ decreases during CA. (a) representative CA-28°C study. (b) Average PCr/tCr_ave_ during circulatory arrest plotted versus surgery parameters. Statistical differences were computed using two-sided paired *t*-tests. ACP studies showed no statistical PCr/tCr_ave_ differences with temperature, hence are plotted together. (c) Linear regression results for average PCr/tCr during CA versus loss of ΔNAA/tCr_ave_.

**FIGURE 6 |:**
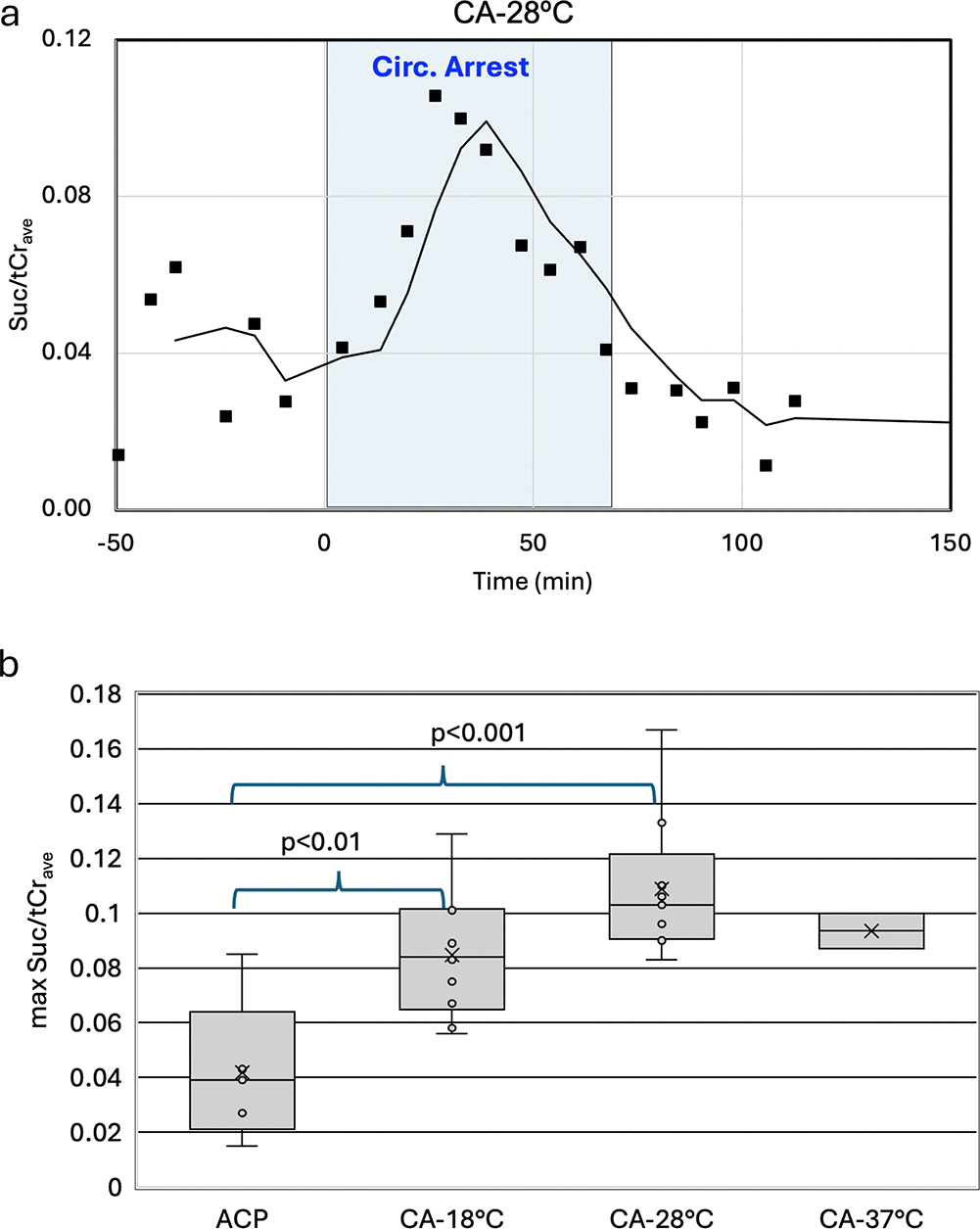
Maximum Suc/tCr_ave_ during CA. (a) Representative CA-28°Cstudy.(b) Maximum Suc/tCr_ave_ during CA plotted versus surgery parameters. Statistical differences were computed using two-sided paired *t*-tests. ACP studies showed no statistical Suc/tCr_ave_ differences with temperature, hence are plotted together. Linear regression results for maximum Suc/tCr_ave_ versus loss of NAA/tCr_ave_ were not statistically significant (*p* = 0.09).

**FIGURE 7 |:**
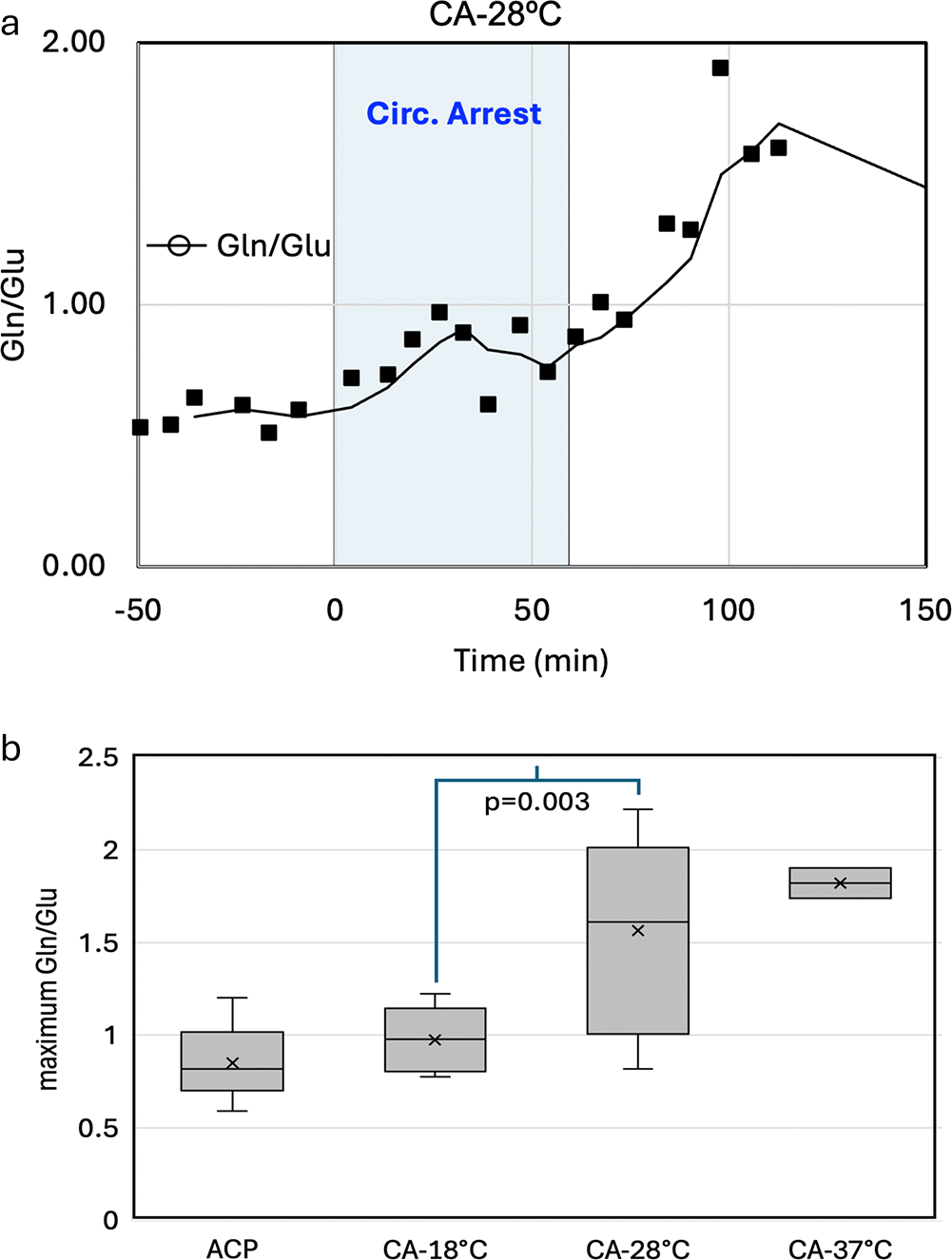
Gln/Glu increases post-circulatory arrest.(a) representative CA-28°C study. (b) Maximum Gln/Glu values occurring post-restart of pump as a function of surgical parameters. Statistical differences were computed using two-sided paired *t*-tests. ACP studies showed no statistical Gln/Glu differences with temperature, hence are plotted together. Linear regression results for maximum Gln/Glu versus loss of NAA/tCr_ave_ were not statistically significant (*p* = 0.35).

As shown in Figure 4a, there was a strong correlation between ΔNAA/tCr_ave_ loss and brain Glc/tCr_ave_ levels immediately prior to CA (*p*=0.008). In addition, blood glucose levels obtained from venous blood samples collected immediately prior to CA were highly correlated with ^1^H-MRS-measured brain Glc/tCr_ave_ (*p<*0.001, Figure 4b). In contrast, elevated brain Lac/tCr_ave_ levels (as assessed by peak values post-CA), while a potential concern with elevated blood glucose levels, did not show any significant correlation with ΔNAA/tCr_ave_. For the CA studies, average PCr/tCr_ave_ levels during CA decreased with increasing hypothermic temperatures (Figure 5b). Loss of NAA/tCr_ave_ was significantly correlated with average PCr/tCr_ave_ levels during CA (*p*=0.0034, see Figure 5c). Although maximum Suc/tCr_ave_ levels during CA increased with hypothermic temperature (Figure 6), correlation between maximum Suc/tCr_ave_ levels at the end of CA and ΔNAA/tCr_ave_ did not reach statistical significance (*p*=0.09). Similarly, elevated brain Gln/Glu ratios post-CA (which are driven by elevated blood ammonia (NH_3_) levels [49]), increased with increasing CA temperatures, (Figure 7b), but the correlation with loss of NAA/tCr_ave_, was not statistically significant (*p*=0.35, see Figure 7c).

To investigate the relative contributions of these four ^1^H-MRS-detectable metabolic ratio metrics, we performed a multivariate regression with ΔNAA/tCr_ave_ as the output and brain Glc/tCr_ave_ immediately prior to CA, average PCr/tCr_ave_ during CA, maximum Suc/tCr_ave_ (which occurred at the end of CA), and post-CA Gln/Glu levels as predictors. The results from the multivariate linear regression analysis are given in Table 2, which show that both pre-CA brain Glc/tCr_ave_ and average PCr/tCr_ave_ during CA were significant predictors of loss of NAA/tCr_ave_ (*p*=0.001 and *p*=0.024 respectively). The Suc/tCr_ave_ and Gln/Glu measures were not found to be statistically significant in this regression analysis.

## 4 | Discussion

Understanding how different surgical approaches affect patient outcomes is essential for developing safer protocols for neonates with congenital heart disease, who face a greater risk of neurodevelopmental delays from CPB procedures. The greater losses in NAA/tCr_ave_ during the CA surgeries compared to ACP correlate with the brain’s increased susceptibility to neuronal injury when perfusion is halted. Strong correlations between NAA/tCr_ave_ loss (as a marker of acute neuronal damage) and both pre-CA brain Glc/tCr_ave_ levels and PCr/tCr_ave_ depletion during CA suggest that reduced ATP availability may significantly contribute to neuronal injury during neonatal CPB surgeries using complete circulatory arrest. Neither reperfusion injury [40], here assessed by elevated Suc/tCr_ave_ during CA, nor ammonia toxicity [48, 58], assessed by elevated post-CA Gln/Glu ratios, was found to correlate with loss of NAA/tCr_ave_.

Glc can be a challenging metabolite to robustly measure in vivo using ^1^H MRS. We didn’t attempt to quantify the Glc from its downfield anomeric proton due to overlap with the water powder pattern from deoxyHb during CA [59, 60]. Despite the spectral overlap, the quantified Glc/tCr_ave_ levels at baseline for all studies strongly correlated with the various starting blood glucose levels (as determined from direct blood glucose assays) and Glc/tCr_ave_ reduction was consistently observed during circulatory arrest.

In addition to brain Glc, the separate quantification of Cre and PCr signals revealed that the loss of PCr during CA, along with the observed NAA losses, supports the energy failure hypothesis [38, 39]. Although the in vivo spectra of PCr and Cre are significantly overlapped, given sufficiently narrow linewidths and accurate measurements of the 3.0 ppm and 3.9 ppm resonances in the PCr and Cre LCModel basis sets, the robust separation of these metabolites is indeed possible at 3T [56]. Here conversion of PCr to Cre during circulatory arrest and PCr/tCr_ave_ recovery during the recovery period was consistently observed for each study (see, for example, Figure 5a).

Although there is extensive literature on the effects of blood Glc levels on CPB surgery outcomes, there are still ongoing investigations focused on determining the optimal preoperative and postoperative blood Glc levels [61]. Multiple studies suggest that high blood Glc levels before or after surgery can be detrimental to patient outcomes [61, 62]. However, the bulk of this literature is likely not directly relevant to the blood Glc manipulation used in our study. Specifically, elevated blood Glc was only transiently induced in the short 45–50min preparatory period immediately prior to circulatory arrest and restored to normal levels upon reperfusion. In a study about Glc-associated alterations in ischemic brain metabolism in neonatal piglets, Laptook et al. suggested that during partial brain ischemia, an elevated plasma Glc may be beneficial for maintaining high-energy phosphates, provided the associated brain acidosis is not deleterious to neonatal brain tissue [63]. Consistent with this, we found no significant correlation between brain Lac/tCr_ave_ and NAA/tCr_ave_ levels.

There however are several limitations with the reporting MRS-detected metabolic changes as ratios relative to total creatine. Bias or physiological changes in tCr may complicate the interpretation of metabolite changes using tCr as a reference. On an animal-by-animal basis, tCr changed during each exam were relatively stable (e.g., Figure 5a). However, tCr variations at times exceed the %SD reported by LCModel and, hence, limiting the validity of reporting observed metabolic changes in terms of absolute concentrations. Additionally, although experimentally measured temperature-dependent LCModel basis functions were used for spectral quantitation of the targeted metabolites, changes in non-metabolic components (e.g., macromolecule contributions and baseline artifacts) with physiology or brain temperature likely introduce bias into the spectral fitting results. For spectra collected at TR 2000 TE 30, water relaxation at different temperatures can also add uncertainty. Alternative metrics include ratios relative to total water content or % deviation of individual metabolites levels relative to baseline values, but these metrics also suffer from similar limitations.

Furthermore, the causes of brain injury, both acute and long-term, are likely multifactorial. Here, we were able to assess metabolic markers correlating with acute neuronal injury due to energy failure, reperfusion injury, and ammonia toxicity. Other injury mechanisms may be present, but we did not identify corresponding ^1^H-MRS detectable measures. In particular, there may be other brain metabolites highly relevant to CPB surgeries, but with concentrations too low for robust detection via in vivo spectroscopy. Importantly, this report also contains no histology or outcome data directly confirming the presence or absence of neuronal damage, and such findings are the target of future investigations. All animals in this study were euthanized within 2h post CA, so the observed NAA/tCr_ave_ losses may reflect transitory injury rather than long term effects as acute neuronal injury does not necessarily imply irreversible damage.

Neither reperfusion injury [40], here assessed by elevated Suc/tCr_ave_ duringCA,nor ammoniatoxicity [48][58], assessedby elevated post-CA Gln/Glu ratios, was found to correlate with loss of NAA/tCr_ave_ in these studies. This could be due to smaller effect sizes needing a greater number of animals to show significance, or the neuronal damage assessment in these experiments being limited by the short time between the onset of reperfusion injury and the final NAA/tCr_ave_ measurement (~1h post-reperfusion).

## 5 | Conclusions

Acute neuronal injury, as measured by loss of NAA/tCr_ave_, was found in a porcine model of CPB surgery involving complete CA. Decreased NAA/tCr_ave_ was correlated with both brain Glc/tCr_ave_ levels at the start of CA and the average value of PCr/tCr_ave_ during CA. The most important conclusions from this preclinical work are: (1) ACP results in no detectable acute brain injury (as measured by MRS), independent of temperature, and (2) acute brain injury in DHCA protocols can be reduced by using a transiently elevated blood glucose level immediately prior to the start of circulatory arrest (with a rapid return to normal glucose levels during rewarming). As with all preclinical findings, directly translating these results to the clinic is challenging.

## Figures and Tables

**TABLE 1 |:** Animal studies listed by surgical type, target hypothermic brain temperature, and blood glucose levels immediately prior to circulatory arrest.

Surgery type	Target temp (°C)	Blood glucose immediately prior to CA (mg/dL)

ACP	18	118
ACP	28	95
ACP	28	214
ACP	32	227
ACP	32	240
CA	18	119
CA	18	157
CA	18	260
CA	18	282
CA	18	339
CA	18	436
CA	18	609
CA	18	662
CA	18	671
CA	18	700
CA	28	54
CA	28	64
CA	28	159
CA	28	182
CA	28	194
CA	28	197
CA	28	268
CA	28	678
CA	28	700
CA	37	121
CA	37	400

**TABLE 2 |:** Results from multiple regression for predicting loss of NAA/tCr ~1h post-CA (ΔNAA/tCr_ave_), using the predictors of brain Glc immediately prior to start of CA (Brain Glc/tCrave), average PCr during CA (Ave PCr/tCr_aave_), max Suc at end of CA (Max Suc/tCr_ave_), and max Gln/Glu ratio post-CA (Max Gln/Glu). Both Brain Glc/tCr_ave_ and Ave PCr/tCr_ave_ were found to be significant with *p* = 0.001 and *p* = 0.024 respectively.

ΔNAA/tCr_ave_	Coefficient	Std. error	*t*	*p* > |*t*|	95% conf. interval	

Brain Glc/tCr_ave_	0.136	0.034	3.98	**0.001**	0.065	0.207
Ave PCr/tCr_ave_	0.244	0.100	2.44	**0.024**	0.036	0.453
Max Suc/tCr_ave_	−0.271	0.571	−0.47	0.641	−1.459	0.918
Max Gln/Glu	0.029	0.040	0.72	0.477	−0.054	0.112
Constant	−0.304	0.075	−4.03	0.001	−0.461	−0.147

*Note:* Bold value indicate significant values.

## Data Availability

The data that support the findings of this study are available on request from the corresponding author. The data are not publicly available due to privacy or ethical restrictions.
